# The DNA methyltransferase inhibitor decitabine blunts the response to a high-animal fat and protein diet in mice

**DOI:** 10.1016/j.jlr.2024.100586

**Published:** 2024-06-26

**Authors:** José de Jesús Flores-Sierra, Magaly del Rosario Muciño-Arellano, Gloria del Carmen Romo-Morales, Jaime Eduardo Sánchez-Palafox, Viridiana Abigail Correa-Navarro, Dannia Colín-Castelán, Victoriano Pérez-Vázquez, Rubén Rangel-Salazar, Rafael Rivera-Bustamante, Carmen de la Rocha, Dalia Rodríguez-Ríos, Diana Lilia Trejo-Saavedra, Jorge Molina-Torres, Enrique Ramírez-Chávez, Nancy Shyrley García-Rojas, Robert Winkler, Gertrud Lund, Silvio Zaina

**Affiliations:** 1 Division of Health Sciences, Department of Medical Sciences, Leon Campus, University of Guanajuato, Leon, Mexico; 2 Tecnológico Nacional de México/ITS de Purísima del Rincón, Purísima del Rincón, Guanajuato, Mexico; 3 Department of Genetic Engineering, CINVESTAV Irapuato Unit, Irapuato, Mexico; 4 Department of Biotechnology and Biochemistry, CINVESTAV Irapuato Unit, Irapuato, Mexico; 5 Unit for Advanced Genomics, CINVESTAV, Irapuato, Mexico

**Keywords:** dietary fat, liver, mitochondria, muscle, nutrition

## Abstract

Increasing evidence hints that DNA hypermethylation may mediate the pathogenic response to cardiovascular risk factors. Here, we tested a corollary of that hypothesis, that is, that the DNA methyltransferase inhibitor decitabine (Dec) ameliorates the metabolic profile of mice fed a moderately high-animal fat and protein diet (HAFPD), a proxy of cardiovascular risk–associated Western-type diet. HAFPD-fed mice were exposed to Dec or vehicle for eight weeks (8W set, 4–32/group). To assess any memory of past exposure to Dec, we surveyed a second mice set treated as 8W but HAFPD-fed for further eight weeks without any Dec (16W set, 4–20/group). In 8W, Dec markedly reduced HAFPD-induced body weight gain in females, but marginally in males. Characterization of females revealed that Dec augmented skeletal muscle lipid content, while decreasing liver fat content and increasing plasma nonesterified fatty acids, adipose insulin resistance, and—although marginally—whole blood acylcarnitines, compared to HAFPD alone. Skeletal muscle mitochondrial DNA copy number was higher in 8W mice exposed to HAFPD and Dec, or in 16W mice fed HAFPD only, relative to 8W mice fed HAFPD only, but Dec induced a transcriptional profile indicative of ameliorated mitochondrial function. Memory of past Dec exposure was tissue-specific and sensitive to both duration of exposure to HAFPD and age. In conclusion, Dec redirected HAFPD-induced lipid accumulation toward the skeletal muscle, likely due to augmented mitochondrial functionality and increased lipid demand. As *caveat*, Dec induced adipose insulin resistance. Our findings may help identifying strategies for prevention and treatment of lipid dysmetabolism.

A wealth of evidence shows that DNA hypermethylation accompanies the natural history of CVD, from exposure to CVD risk factors to the manifestation of atherosclerosis. The data are consistent whether global—that is, total methylcytosine, bulk DNA methylation-sensitive restriction sites—or locus-specific DNA methylation is determined. High-fat diets (HFDs) induce DNA hypermethylation in the liver, placenta, and skeletal muscle in humans and rodent models (1, 2, 3, 4, 5, 6) and increase DNA methyltransferase expression (7, 8). The same overall response is obtained by exposing human adipose tissue or cultured cells to selected dietary FAs or atherogenic lipoprotein fractions (9, 10, 11). As *caveat*, recent data show that loss of DNA methyltransferase expression in hematopoietic cells actually exacerbates the response to an HFD in mice, suggesting a degree of cell specificity (12). DNA hypermethylation is associated with CVD risk in humans (13, 14, 15). Importantly, the DNA methylation inhibitor azacytidine was singled out as a prominent inducer of atheroprotective gene expression in a large-scale molecular screen (16). Moreover, the trend toward DNA hypermethylation is maintained in the atherosclerotic vascular wall. The formation of arterial neointima, a proxy of the atheroma, is accompanied by DNA hypermethylation (17, 18). Proatherogenic blood flow induces DNA hypermethylation in the endothelium (19). Decreased expression of enzymes involved in DNA demethylation accompanies human peripheral artery disease and transplant vasculopathy (20, 21). Experimentally, independent approaches to biochemically inhibit DNA methylation or overexpress enzymes involved in DNA demethylation resulted in the mitigation of atherosclerosis in animal models (22, 23, 24, 25, 26, 27). The literature on the DNA methylome of the atheroma has been recently reviewed (28).

The above-mentioned evidence points to DNA hypermethylation as proatherogenic, yet to our knowledge the causal contribution of DNA hypermethylation to the metabolic alterations induced by exposure to CVD risk factors before the onset of atherosclerosis has not been addressed experimentally. In the present study, we challenged a mouse model of moderately high-animal fat and protein diet (HAFPD) with decitabine (Dec), a DNA methylation inhibitor. If the hypothesis that DNA hypermethylation is a causal mediator of the metabolic effects of HFD is correct, Dec is expected to reduce HAFPD-induced body weight (BW) gain. As secondary outcomes, Dec is expected to ameliorate metabolic variables involved in BW gain and energy expenditure. We also assessed whether any memory of Dec treatment is retained after withdrawal. We discuss the results in the light of current literature on metabolic disease, CVD, and epigenetics.

## Materials and methods

### Mice

Animal procedures were reviewed and approved by the ethical committee of the Department of Medical Sciences and by the University of Guanajuato institutional committee for ethics in research (approval number CIBIUG-A32-2017 and CEPIUG-A07-2023). The study was entirely or partly repeated four times during 2014–2015, 2017–2018, 2021, and 2023–2024, by three different supervised trainees who performed most day-to-day animal and laboratory experimental procedures, using four different batches of HAFPD and Dec. Each experimental procedure was performed at least twice, except for the determination of whole blood acylcarnitines, insulin and nonesterified fatty acids (NEFAs), which were performed once.

HAFPD was Purina® Pro Plan® puppy small breed with Optistart® plus (see supplemental Table S1 for HAFPD composition). HAFPD is a commercial diet containing 20% fat, 32% protein, designed for fast-growing young animals. This corresponds to 4-fold higher fat and ∼34% higher protein content than chow (LabDiet no. 5001; supplemental Table S1). Fat and protein are mainly from egg and poultry in HAFPD but of balanced plant, fish, and porcine origin in chow. As percent of total FA (determined essentially as in (29)), compounded saturated and polyunsaturated FA are higher and lower in HAFPD than chow, respectively. The main saturated FA are palmitic and stearic acid (C16:0 and C18:0, respectively), which modestly differ between HAFPD and chow (+6.8% and +58.2% in HAFPD, respectively), but the 4.5-fold excess of C15:0, a component of food of animal origin is notable; other indicators of animal origin are branched chain FAs, which are present only in HAFPD and likely derive from animal adipose tissue or rumen (30, 31). Also, the *trans* FA elaidate (C18:1) is 36.3% higher in HAFPD than chow (supplemental Table S1). These differences are even more prominent if the 4-fold excess of total fat in HAFPD compared to chow is considered. Therefore, HAFPD is a proxy of Western-type high-animal fat and protein diet (see for example the average diet in the US population, a high cardiometabolic risk population; source: USDA, www.ars.usda.gov/nea/bhnrc/fsrg) (32). Fat content of HAFPD is moderately high compared to diets frequently employed in mouse studies of metabolic diseases, nonetheless its similarity to human Western-type diet and ability to induce atherosclerosis in hyperlipidemia-prone mice have been acknowledged (33, 34). We employed the atherosclerosis-resistant wild-type C57BL/6 mouse strain, a suitable model of CVD risk–enhancing diet with no concomitant histologically obvious atherosclerosis. The nucleoside analog Dec (2′-deoxy-5-azacytidine) was chosen based on the rationale that it is a stable, water-soluble, easy to administer compound. Dec and the chemically similar azacytidine are antitumor drugs used against a variety of cancers in clinical trials and in therapy, but Dec shows a higher specificity for DNA demethylation (35, 36).

**Fig. 1:**
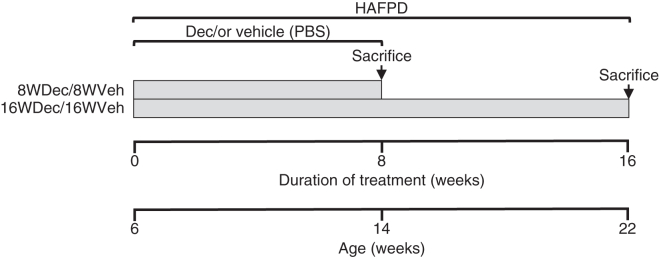
Synopsis of study design. Six-month-old mice were exposed to high-animal fat and protein diet (HAFPD) for 8 or 16 weeks, in addition to decitabine (Dec) or vehicle (Veh) during the first 8 weeks (8WDec, 8WVeh, 16WDec, or 16WVeh, respectively). Mice of 8W and 16W experimental groups were sacrificed and analyzed at week 8 or 16. The same experimental protocol but with chow feeding instead of HAFPD was conducted in parallel (not represented in the figure). Sample sizes for each specific analysis are indicated in the corresponding figure or table.

HAFPD was administered ad libitum. Dec (1 μg/g; Sigma-Aldrich no. A3656) in PBS or vehicle (Veh) alone were administered weekly by subcutaneous injection. Dec dosage was the same as the azacytidine dosage that yielded effective DNA demethylation without any gross adverse effect in mice (37). Absolute Dec dose (μg) is shown in supplemental Table S2. A schematic view of the study is shown in Figure 1. In the experiments conducted in 2014–2015, 2017–2018, and 2021, six-week-old mice were randomly divided into two sets. The first set received HAFPD and either Dec or Veh for a period of eight weeks (referred to as 8WDec or 8WVeh groups, respectively, and collectively as 8W). The sample size of each determination performed in this study is indicated in the respective figure or table legend. 8W were sacrificed at the eight-week treatment termination. 8W therefore tested the effects of Dec when coadministered with HAFPD. The second set underwent the same protocol as 8W but was further exposed to HAFPD for eight additional weeks without any Dec: this set is referred to as 16WDec (exposed to HAFPD and Dec in the first 8-week treatment) or 16WVeh (exposed to HAFPD alone in the first 8-week treatment), and collectively as 16W. 16W were sacrificed at the 16-week treatment termination. 16W therefore tested the memory of past exposure to Dec. A similar experimental scheme was conducted with chow feeding instead of HAFPD. In the experiments conducted in 2023–2024 to determine glucose, insulin and NEFA, 8W and 16W mice were the same, as blood was obtained from the tail tip at the eighth week and sacrifice was performed at the 16th week of the protocol. Mice were sacrificed by decapitation under anesthesia with isoflurane, following an overnight fasting with free access to water. Blood was collected in EDTA-coated tubes and plasma, blood cell pellet, or whole blood was frozen in aliquots. Organs were dissected, immediately immersed in RNAlater (Thermo Fisher Scientific), and stored at −80ºC. Skeletal muscle was the *biceps femoris*. The adipose tissue was epididymal.

### Somatometry and food intake

BW and food intake, calculated as difference between supplied and recovered food, were determined weekly in the early morning. Body length, tip of nose-to-base of tail distance, and tibial length were determined at sacrifice.

### DNA methylation

Global DNA methylation was determined by either of two assays. The ELISA-based MethylFlash Methylated DNA 5-mC Quantification Kit, Colorimetric (Epigentek) was used following the manufacturer's instructions. The assay determines total methylcytosine. The second assay yields the methylation status of the bulk of genomic restriction sites for *Hpa*II, a methylation-sensitive endonuclease. The assay was carried out as published with minor modifications that were recently published by us (38, 39).

### Circulating metabolism-related factors

Plasma lipoproteins, triglycerides, and cholesterol were determined at the Department of Medical Sciences' Clinical Laboratory by standard methods. Glucose was determined with the Accu-Chek Performa system (Roche). Total adiponectin and insulin were determined by ELISA (adiponectin: 47-ADPMS-E01; insulin: 80-INSMSU-E01; both from ALPCO Immunoassays) following manufacturer's instructions. The homeostasis model assessment of insulin resistance (HOMA-IR) was determined as fasting plasma insulin (pM) × fasting plasma glucose (mM)/22.5, as reported in mice (40). Adipose tissue insulin resistance (adipose-IR) was calculated as fasting plasma insulin (pM) × fasting plasma NEFA (mM) (41).

NEFA were determined in 50 μl plasma. Samples were lyophilized and NEFA were extracted with isooctane and derivatized in situ. NEFA present in the isooctane-extracted lipid fraction were selectively derivatized by silylation with N,O-Bis(trimethylsilyl)trifluoroacetamide, which converts the accessible hydroxyl group of NEFA to trimethylsilyl ether and does not modify esterified FA (42). Extraction and silylation were simultaneously performed with 100 μl isooctane, 20 μl N,O-Bis(trimethylsilyl)trifluoroacetamide, and 1% trimethylchlorosilane (all from Sigma-Aldrich) with agitation in a Thermomixer comfort (Eppendorf) at 80°C for 30 min. After cooling, samples were centrifuged at 3,000 rpm for 5 min. The supernatant was analyzed by GC-MS for the determination of total FA methyl esters as previously reported (29). Pure heptadecanoic acid (Sigma-Aldrich) was used as standard for NEFA quantification.

Whole blood acylcarnitines were determined by direct liquid introduction MS/MS. Six microliters blood internal standard solution and methanol (75 mg sample per microliter methanol ratio) were added to weighed tubes containing lyophilized EDTA-treated peripheral blood (100 μl original volume). Blood internal standard contained 7.6 μM palmito-yl-L-carnitine-N-methyl-d3-HCl, 3.8 μM of each myristoyl-L-carnitine-N, N, N-methyl-d9-HCl, octanoyl-L-carnitine-N-methyl-D3-HCl, isovaleryl-L-carnitine-N, N, N-methyl-d9-HCl, butyryl-L-carnitine-d3-HCl, propionyl-L-carnitine-d3-HCl, 19 μM acetyl--L-carnitine-N-methyl-d3-HCl, and 76 μM L-carnitine-trymethyl-d9-HCl. Samples were mixed in an orbital shaker for 30 min at 850 r.p.m. Following centrifugation at 2,000 *g* for 5 min, 200 μl supernatant was transferred to 96-well plates. Samples were dried at 50ºC for 20 min, and 100 μl 3M HCl in methanol was quickly added, followed by incubation at 50ºC for 15 min, cooling for 2 min, and evaporation to dryness at 50ºC for 20 min. Samples were resuspended in 1 ml methanol: deionized water 85:15 ratio solution. An Agilent 1260 series UHPLC system (Palo Alto, CA) coupled with an AB Sciex QTRAP® 4000 mass spectrometer (Concord, ON) was used for all online LC/direct liquid introduction-MS/MS analyses. Red PEEK tubing connected the LC system with the MS system. The controlling software for the sample analysis was Analyst® 1.5.3. The data analysis was done using data and OrgMassSpecR libraries on the statistical computing and graphics programming language R. A UHPLC autosampler was connected directly to the QTRAP® 4000 MS Turbo V™ ion source via red PEEK tubing. The solvent used for sample dilution was also used as the mobile phase. The flow rate was set as follows: t = 0 min, 0.03 ml/min; t = 1.6 min, 0.03 ml/min; t = 2.4 min; 0.2 ml/min; t = 2.8 min, 0.2 ml/min; and t = 3.0 min, 0.03 ml/min. Next, 20 μl of each sample solution was injected into the QTRAP® 4000 MS for analysis. The QTRAP® 4000 *MS* was set to a positive ESI mode with multiple reaction monitoring scanning to analyze acylcarnitines. The IonSpray voltage was set at 5500 V for the positive mode, and the ion source temperature was set at 200°C. The CUR, GAS1, GAS2, and CAD were set at 20, 40, 50, and medium, respectively. The EP and CXP were set at 10 V and 15 V separately. Likewise, the DP, CE, Q1, and Q3 were optimized and set individually for each analyte and the internal standard. Acylcarnitines will be indicated as standard annotation of the corresponding FA, number of carbons, degree of saturation and hydroxylation if any, followed by a C for carnitine (example: C16C for palmitoylcarnitine).

### Tissue total lipid content

Fresh tissue fragments (25–30 mg) were briefly dried with blotting paper, weighed, and fat was extracted with chloroform as described (29). Total fat was calculated as the percent decrease in fragment weight after extraction. That value is therefore corrected for the initial weight of the tissue fragment and, by extrapolation, for organ weight. Whole liver color as proxy of hepatic fat content was quantified using the ImageJ software (https://imagej.net/software/imagej/).

### Mitochondrial DNA copy number

Fifteen nanograms SYBR Green–quantified genomic DNA were amplified with mitochondrial DNA-specific primers as described (43). All primers used in this study were synthesized by T4OLIGO (Mexico). PCR products were quantified by SYBR Green against a reference curve built with DNA of known concentration. PCR runs were deemed acceptable if amplification of 15 and 7.5 ng of the same randomly chosen experimental sample yielded PCR products with 2:1 amount ratio.

### Gene expression

Total RNA was extracted with QIAzol Lysis Reagent (QIAGEN®, cat. no. 79306). RNA was reverse transcribed with the QuantiTect® Reverse Transcription kit (QIAGEN®, cat. no. 205311). Quantitative gene expression was determined by amplifying cDNA with the QuantiNova SYBR Green PCR kit (QIAGEN®, cat. no. 208054) and gene-specific primers in a Bio-Rad CFX96 cycler, in duplicate. Sequences of primers for *Drp1*, *Fis1*, *Gapdh*, *Mfn2*, optic atrophy 1 (*Opa1*), and *p62* were as reported (44). Expression was calculated relative to *Gapdh* using the 2^ΔCT^ formula. Control amplifications without any cDNA were run to verify lack of primer dimerization or nonspecific amplification.

### Statistics

Individual mouse values from different experiment repeats were pooled into a single sample, and HAFPD and Dec batch were included as categorical (1–4) covariables. The sample size for the determination of the primary outcome (BW) in either gender was larger than the one used in a similar analysis of mouse BW (45). Sample size in secondary outcome determinations were dictated by available samples in each specific case. Percentage values were logit-transformed. Unless stated otherwise, ANOVA with Scheffé's post hoc was used in all comparisons.

## Results

### Dec elicits expected effects on global DNA methylation

The ability of Dec to elicit DNA hypomethylation in our model was assessed by measuring global DNA methylation with two independent techniques, that is, total methylcytosine or bulk *Hpa*II site methylation quantification, in liver, skeletal muscle, and epididymal adipose tissue (supplemental Fig. S1). Dec consistently elicited a significant hypomethylation of liver DNA in 8W, although the extent varied between the two techniques (compare supplemental Fig. S1, *A* and *B*). That variation was expected, as different genomic contexts are surveyed in the two cases. DNA methylation was determined in skeletal muscle and epididymal adipose tissue in 8W and 16W by *Hpa*II site methylation quantification assay. The data indicate that memory of previous exposure to Dec was restricted to the liver, as Dec-induced DNA hypomethylation was stable in that tissue following the eight week withdrawal from Dec but returned to control (Veh) levels in the skeletal muscle and adipose tissue (supplemental Fig. S1, *B–D*).

### Dec decreases BW gain

**Fig. 2:**
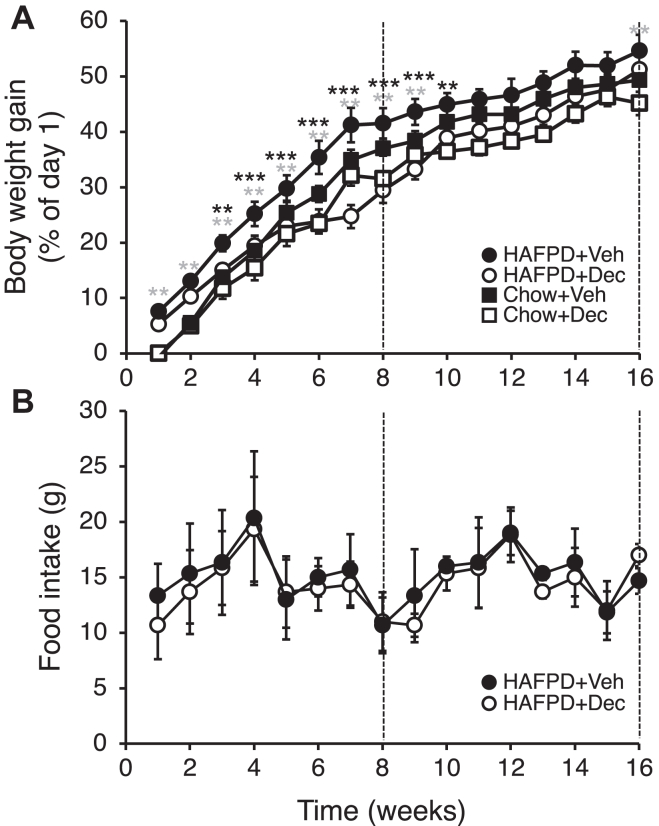
Effects of decitabine on body weight gain and food intake in high-animal fat and protein diet (HAFPD)-fed female mice. The vertical dashed lines mark the time of sacrifice of mice with continuous exposure to Dec for eight weeks (8W set, left-hand dashed line) or with Dec withdrawal at week 8 (16W set, right-hand dashed line). A: body weight gain. HAFPD-fed groups: n = 32/group in 8W, that is, 2014–2015, 2017–2018, and 2021 data for 12 8W and 10 16W combined; additionally, n = 10 from 2023–2024 data; n = 20/group for 16W, that is between week 8 and 16, data from all years combined. Chow-fed groups: n = 18 in 8W, that is, 2021 data for four 8W and four 16W combined; additionally, n = 10 from 2023–2024 data; n = 14/group for 16W, that is between week 8 and 16, data from all years combined. Asterisks above time points indicate significant differences between Dec-exposed and Veh-exposed HAFPD-fed mice (black asterisks) or between Veh-exposed HAFPD-fed and chow-fed mice (gray asterisks). B, food intake (n as in A). For other symbols see legend of Figure 1. Data are mean ± SEM. ∗∗*P* < 0.01. ∗∗∗*P* < 0.001. Kruskal–Wallis test and ANOVA Scheffé’s post hoc.

We tested the ability of Dec to modify BW gain of male or female mice fed HAFPD for 8 and 16 weeks. The data revealed a significant sexual dimorphism in the response to Dec: in males, Dec significantly reduced BW gain within a narrow time frame of HAFPD exposure, that is, between weeks 5 and 7 (supplemental Fig. S2); in females however, Dec exposure led to a significant reduction in BW gain between week 3 and 8 of HAFPD feeding, and during a limited time period following Dec withdrawal (*i*.*e*., between week 9 and 10 of HAFPD) compared to the time-matched Veh-exposed females, indicating a transient effect of Dec (Fig. 2*A*). The transient effect of Dec could not be explained by HAFPD-induced catch-up after Dec withdrawal, but rather coincided with BW gain decrease after week 7–8, when BW gain of 16WVeh and chow-fed mice realigned after a phase of significantly higher BW gain in HAFPD-fed mice relative to chow-fed controls (Fig. 2*A*). Based on examination of general health—mobility, fur appearance, food intake (see below)—we conclude that Dec did not elicit any macroscopic adverse effect on mouse welfare. Based on the comparatively weak response of male mice, we carried out all subsequent steps of the study in female mice, which are henceforth referred to as "mice."

A candidate underlying mechanism of the Dec-induced decrease of BW gain in HAFPD-fed mice was anorexigenic activity. That possibility was ruled out, as Dec did not induce any significant decrease of food intake (Fig. 2*B*).

### Effects of exposure to Dec on somatometric or plasma metabolic variables

**Fig. 3:**
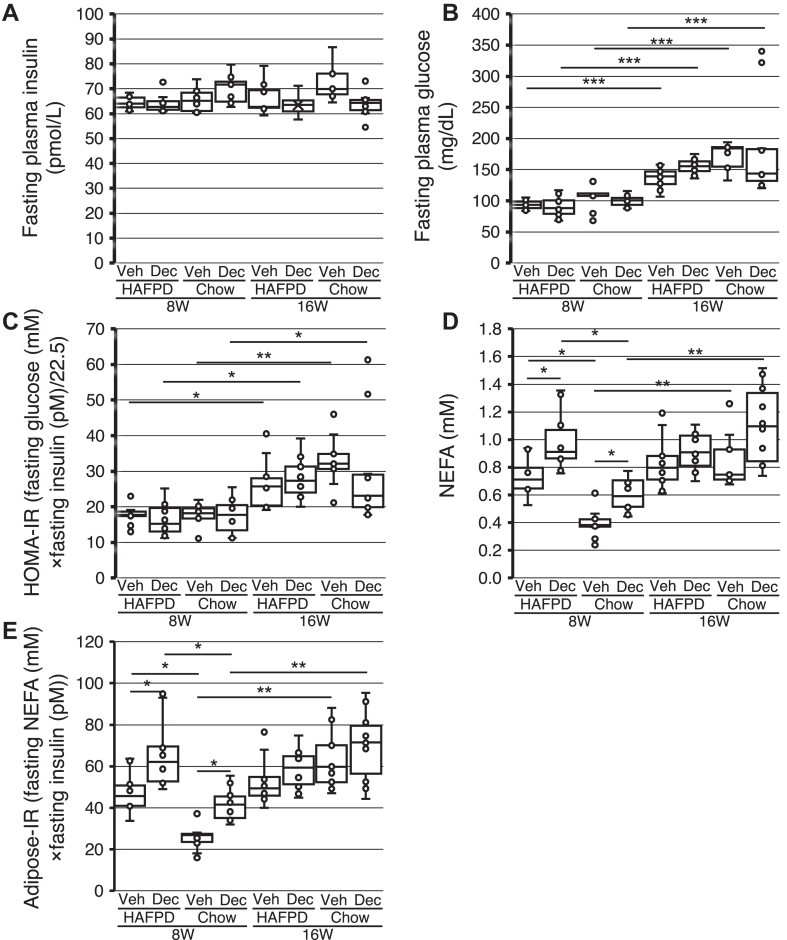
Effects of decitabine on fasting plasma glucose, insulin, and insulin resistance in high-animal fat and protein diet (HAFPD)-fed female mice. Box and whisker plots of data from HAFPD-fed or chow-fed mice exposed to vehicle or decitabine (Veh or Dec, respectively). For other symbols see Figure 1 legend. Asterisks above boxes indicate significant differences between corresponding 8W and 16W groups or within 8W or 16W. ∗*P* < 0.05; ∗∗*P* < 0.01; and ∗∗∗*P* < 0.001. ANOVA Scheffé’s post hoc. Glucose: n = 22 in HAFPD-fed 8W, n = 20 in HAFPD-fed 16W. All other measurements: n = 10/group, except n = 9 in chow-fed 16WVeh. HOMA-IR, homeostasis model assessment of insulin resistance; adipose-IR, adipose tissue insulin resistance.

To understand the mechanisms underlying the observed responses to Dec, we obtained somatometric data, that is, body and tibial length. Our rationale was that although exposed mice were adults, it could not be ruled out that Dec elicited any reprogramming of cell lineages involved in skeletal growth. Dec did not significantly affect skeletal growth (Table 1). Similarly, circulating factors involved in the regulation of body fat mass and lipid metabolism—lipids, lipoproteins, adiponectin, and insulin—were not significantly different between Veh and Dec groups in 8W or 16W, whether HAFPD-fed or chow-fed (Table 1 and Fig. 3*A*). Lack of statistical power does not explain those results, as all parameters changed by 10% or less between 8WDec and 8WVeh except for triglycerides, which displayed a high intragroup variability. However, glucose was significantly higher in HAFPD-fed 16W than the corresponding 8W, irrespective of exposure to Dec (highest *P* = 5.9 × 10^−12^) (Fig. 3*B*). To assess whether the observed change in glucose was due to length of HAFPD treatment or age-related, we analyzed chow-fed mice exposed to Dec or Veh by the same protocol used for HAFPD-fed mice. A similar glucose profile was observed in chow-fed mice, and no significant difference was observed between HAFPD-fed and chow-fed mice at identical time points. Thus, the data revealed an age-dependent increase of glycemia. Mean glucose remained below the accepted 250 mg/dl threshold for mouse hyperglycemia in all groups (46). To determine whether insulin resistance is involved in the responses to Dec, we measured HOMA-IR and adipose-IR. HOMA-IR was not affected by HAFD or Dec, but was significantly higher in 16W irrespective of diet or treatment, thus reflecting the age-related increase in glucose (highest *P* = 0.033) (Fig. 3*C*). Adipose-IR is calculated using insulin and NEFA (41). A total of 11 NEFA were detected (raw data are shown in supplemental Table S3). NEFA data for the different groups are shown in Figure 3*D*. HAFPD increased NEFA compared to chow in 8W by ∼80%; Dec per se increased NEFA by ∼30% in either group, compared to Veh. By contrast, an age-related elevation in NEFA was observed in 16W. Adipose-IR followed the same trend in 8W and 16W (Fig. 3*E*). Consistent with the differential effects of HAFPD or Dec on HOMA-IR and adipose-IR, the two indexes were not significantly correlated in the overall sample (r = 0.18, *P* = 0.104, n = 79).

**Table 1:** Somatometric and selected plasma metabolic parameters

	8WVeh	8WDec	16WVeh	16WDec
Body length (cm)	8.3 ± 0.5 (12)	8.3 ± 0.4 (12)	9.8 ± 0.6 (10)	9.6 ± 0.7 (10)
Tibial length (cm)	2.0 ± 0.1 (12)	2.1 ± 0.2 (12)	2.7 ± 0.3 (10)	2.6 ± 0.3 (10)
Triglycerides (mg/dl)	101.6 ± 18.8 (12)	140.6 ± 23.5 (12)	177.0 ± 20.6 (10)	156.8 ± 41.2 (10)
Cholesterol (mg/dl)	143.0 ± 13.7 (12)	140.4 ± 18.0 (12)	192.2 ± 27.5 (10)	171.0 ± 16.0 (10)
VLDL cholesterol (mg/dl)	16.3 ± 1.2 (12)	17.2 ± 2.1 (12)	18.3 ± 1.5 (10)	18.2 ± 2.3 (10)
LDL cholesterol (mg/dl)	6.8 ± 4.1 (12)	2.9 ± 3.0 (12)	6.9 ± 4.0 (10)	3.9 ± 3.2 (10)
HDL cholesterol (mg/dl)	71.0 ± 6.0 (12)	80.8 ± 12.9 (12)	68.0 ± 6.6 (10)	81.2 ± 13.9 (10)
Adiponectin (μg/ml)	29.9 ± 3.0 (10)	25.9 ± 2.8 (10)	31.0 ± 3.1 (10)	29.3 ± 14.9 (10)

8WVeh and 8WDec: mice fed with high-animal fat and protein diet or chow, either combined with decitabine (Dec) or vehicle only (Veh) for eight weeks. 16WVeh and 16WDec: mice treated as the 8W groups, but fed with high-animal fat and protein diet or chow for further eight weeks, without any Dec (i.e., exposed to vehicle only). Data are shown as mean ± SEM (n).

Dec, decitabine; Veh, vehicle.

### Effects of exposure to Dec on organ weight, lipid accumulation, and mitochondrial homeostasis

In the light of the Dec-induced increase in plasma NEFA and to further understand the effects of Dec on BW gain, we tested the hypothesis that Dec modulates organ weight, lipid content, and mitochondrial DNA content, a proxy of mitochondrial abundance, in the liver and skeletal muscle. We complemented those data by determining the expression of key mitochondrial homeostasis genes in the skeletal muscle and whole blood acylcarnitines.

**Fig. 4:**
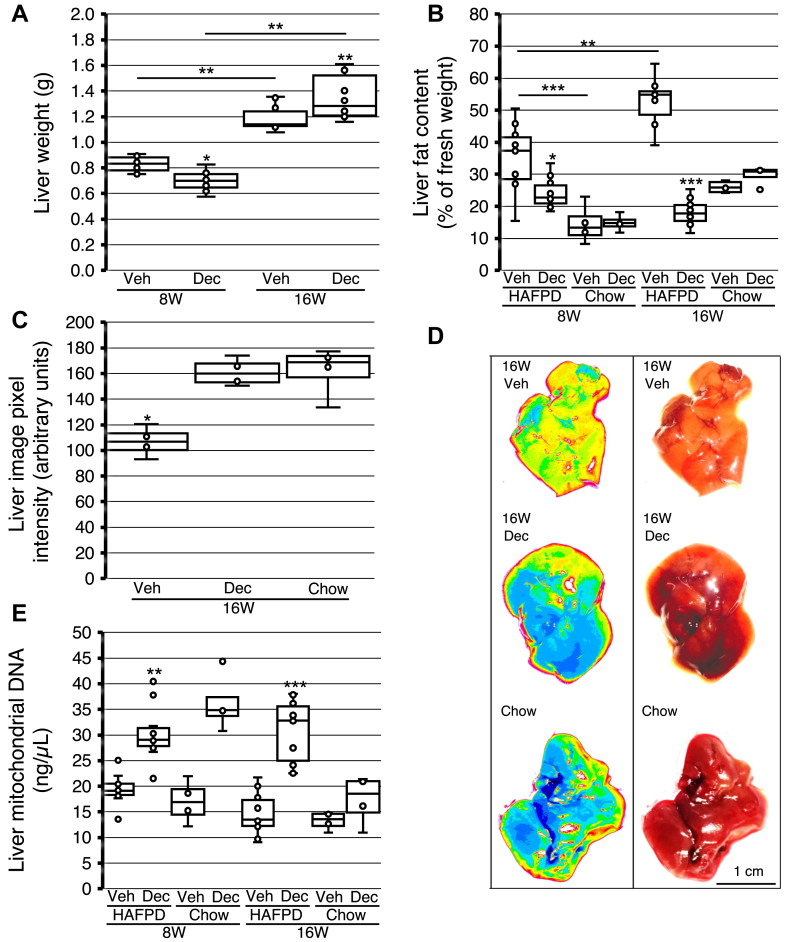
Effects of decitabine on liver weight, fat content, and mitochondrial homeostasis in high-animal fat and protein diet (HAFPD)-fed female mice. Box and whisker plots of data from HAFPD-fed or chow-fed mice exposed to vehicle or decitabine (Veh or Dec, respectively). Asterisks above boxes indicate significance of HAFPD-fed, Dec-exposed mice in comparison with the respective Veh (A, B, E), or with Dec or chow (C), respectively. ∗*P* < 0.05; ∗∗*P* < 0.01; ∗∗∗*P* < 0.001. Kruskal–Wallis and ANOVA Scheffé’s post hoc. For other symbols see Figure 1 legend. A: liver weight in HAFPD-fed mice (n = 10/group). B: liver fat as percentage of chloroform-extracted weight (n = 10/group). C: liver color intensity as proxy of liver fat (n = 4/group) in 16W. Note that pixel intensity is inversely associated with fat content. D: representative livers analyzed in C. Left and right, image-processed, and native liver images. E, mitochondrial DNA content (n = 10/group).

We first assessed the effect of HAFPD either alone or combined with Dec on liver weight. This gross analysis did not include any comparison with chow-fed mice. Duration of exposure to HAFPD alone or age or a combination thereof increased liver weight (*P* = 0.010; compare 8WVeh and 16WVeh in Fig. 4*A*). Dec slightly but significantly decreased liver weight in 8W compared to exposure to HAFPD alone, but Dec withdrawal had the opposite effect (*P* = 0.006 in the comparison between 8WDec and 16WDec, Fig. 4*A*). As indirect validation of our results, we asked whether the significant positive association between liver weight and global DNA methylation that was observed in an independent study, which could be replicated in our model (29). Paired liver weight and *Hpa*II site methylation were directly associated in 8WDec and 16WDec, although significance was modest (r = 0.83, *P* = 0.041 in 8W, and r = 0.82, *P* = 0.045 in 16W; n = 5 in either case), but not in 8WVeh or 16WVeh.

To fully appreciate any effect of HAFPD, Dec or their combination on organ fat content, we compared age-matched and length of treatment-matched chow-fed mice. Liver fat content (weight) data are shown in Figure 4*B*. Chloroform-extractable lipid content was ∼2-fold higher in 8WVeh livers than 8W-matched chow-fed controls (33.2 ± 12.9 against 14.0 ± 6.1%, respectively, *P* = 0.002); the same pattern was observed in 16W (52.1 ± 10.2 against 26.0 ± 5.1%, respectively, *P* = 1.2 × 10^-4^). Liver fat content was significantly increased with length of exposure to HAFPD alone (*P* = 0.003, compare 8WVeh and 16WVeh), but the difference in liver fat between 8W-matched and 16W-matched chow-fed mice was marginally significant (14.0 ± 6.1 and 26.0 ± 5.1%, respectively, *P* = 0.061). Therefore, duration of HAFPD feeding had a predominant effect on liver fat content rather than ageing per se. Dec induced a relatively small but significant decrease in liver fat content in 8W but induced a marked effect in 16W, to levels similar to livers of matched chow-fed mice (18.3 ± 4.6 against 26.0 ± 5.1%, respectively, *P* = 0.206). These observations were replicated when liver digitalized color (pixel intensity) was assessed as proxy of fat content in 16W: 16WDec livers displayed pixel intensity at levels not statistically different from matched chow-fed counterparts but clearly different from 16WVeh (Fig. 4, *C* and *D*). As for liver mitochondrial DNA content, no difference was observed between 8WVeh or 16WVeh and matched chow-fed counterparts (lowest *P* = 0.164). However, mitochondrial DNA content was higher in 8WDec or 16WDec livers relative to 8WVeh or 16WVeh counterparts (Fig. 4*E*). In fact, mitochondrial DNA content and fat content essentially followed opposite patterns when Dec-exposed livers were compared with the respective Veh control, with a clear memory of previous exposure to Dec in either case (compare Fig. 4, *B* and *E*).

### Effects of exposure to Dec on skeletal muscle lipid accumulation and mitochondrial homeostasis

**Fig. 5:**
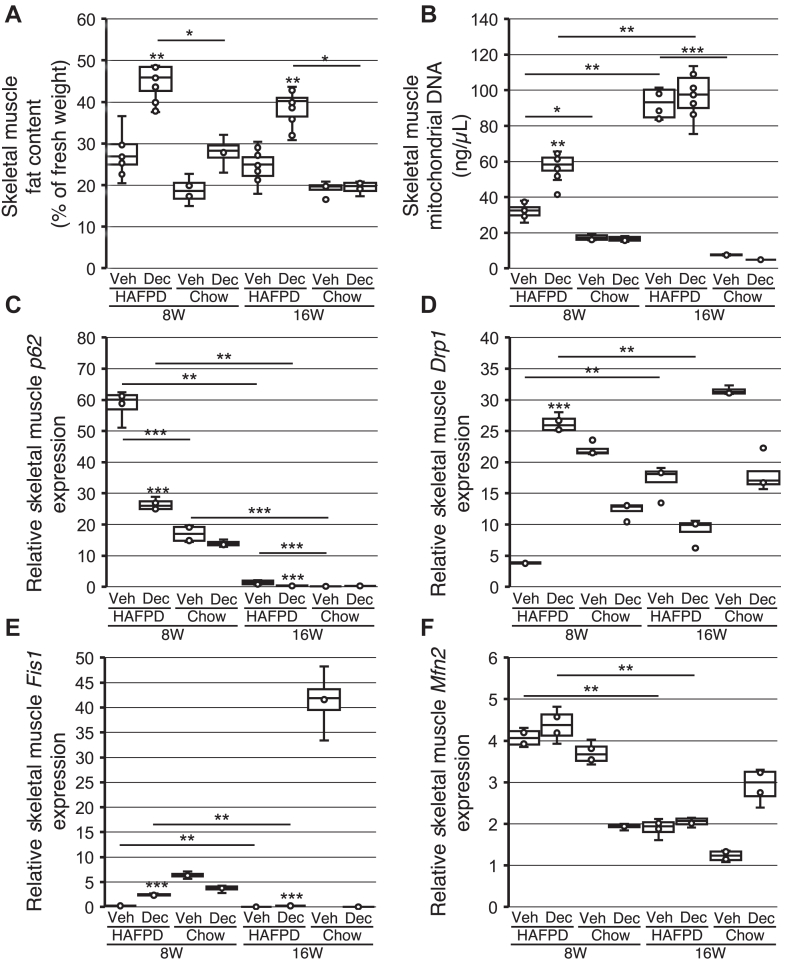
Effects of decitabine on skeletal muscle fat content and mitochondrial homeostasis in high-animal fat and protein diet (HAFPD)-fed female mice. Box and whisker plots of data from HAFPD-fed or chow-fed mice exposed to vehicle or decitabine (Veh or Dec, respectively). Asterisks directly above boxes indicate significance in comparison with the respective Veh. ∗*P* < 0.05; ∗∗*P* < 0.01; and ∗∗∗*P* < 0.001. Kruskal–Wallis and ANOVA Scheffé’s post hoc. A, B: n = 12/group (8W) or n = 10/group (16W). C–F: expression relative to *Gapdh* (n = 4/group).

In the skeletal muscle, the responses to HAFPD or Dec were distinct from the ones observed in the liver. First, lipid content did not differ between 8WVeh and matched chow-fed controls (27.2 ± 6.1 and 18.2 ± 7.9%, respectively, *P* = 0.066); the same pattern was observed in 16W (24.2 ± 4.7 and 18.7 ± 5.7%, respectively, *P* = 0.252). Second, duration of HAFPD feeding did not alter lipid content (*P* = 0.242; compare 8WVeh and 16WVeh in Fig. 5*A*). Third, lipid content was significantly higher in 8WDec than 8WVeh and that pattern persisted in 16W (Fig. 5*A*). In fact, the trends of liver and skeletal muscle fat content were opposite in either 8W or 16W (compare Figs. 4*B* and 5*A*). 8WDec and 16WDec showed a significantly higher skeletal muscle lipid content than matched chow-fed controls (8W: 44.1 ± 6.5 and 28.2 ± 7.5%, respectively, *P* = 0.018; 16W: 38.4 ± 4.3 and 18.9 ± 9.6%, respectively, *P* = 0.019). Mitochondrial DNA content was modestly but significantly higher in 8WVeh skeletal muscle than chow-fed counterparts (32.5 ± 5.5 ng/μl and 17.3 ± 5.4 ng/μl, respectively, *P* = 0.046), but that difference was highly significant in 16W (92.2 ± 24.1 ng/μl in 16WVeh and 7.5 ± 2.4 ng/μl in chow-fed controls, *P* = 8.4 × 10^–9^). The wider gap in mitochondrial DNA content between 16WVeh and chow-fed controls compared to 8W was due to an increase with time of HAFPD feeding (*i.e*, between 8WVeh and 16WVeh) and a concomitant decrease in chow-fed mice over the same period, that is, the component attributable to ageing. 8WDec mitochondrial DNA content was significantly higher than 8WVeh, but 16WVeh and 16WDec were not statistically different (Fig. 5*B*). Skeletal muscle fat content and mitochondrial DNA content followed the same trend in 8W but not in 16W, where prolonged HAFPD feeding increased mitochondrial DNA content, irrespective of any previous exposure (compare Fig. 5, *A* and *B*). Thus, the data revealed a paradoxically similar effect of duration of HAFPD feeding, that is, from 8W to 16W, and of exposure to Dec in 8W in skeletal muscle: either treatment increased both mitochondrial DNA content and lipid accumulation compared to 8WVeh, despite their opposite effects on BW (compare Fig. 5, *A* and *B*). Notably, the adipose tissue mitochondrial DNA content showed the same trend as the skeletal muscle in 8W and 16W, thus suggesting a widespread and experimentally reproducible effect of Dec (supplemental Fig. S3).

### Dec alters mitochondria-related gene expression in skeletal muscle

A possible explanation for the similar effects of either length of feeding with HAFPD or exposure to Dec in 8W on skeletal muscle mitochondrial DNA content is that mitochondrial pools are functionally distinct in the two cases: we hypothesized that feeding with HAFPD induces dysfunctional mitochondria akin to known responses to HFDs in rodent models, whereas Dec increases the number of functional mitochondria, resulting in increased lipid demand; in turn, the latter results in lipid accumulation if the lipolysis rate does not match de novo lipogenesis or the lipid flux rate from the liver or adipose tissue (Fig. 5, *A* and *B*) (47, 48). That phenomenon may resemble the human "athlete's paradox" (49, 50). A balance among autophagy, fission, and fusion is a hallmark of functional mitochondria. Autophagy eliminates damaged mitochondria and maintains mitochondrial functionality (51). Broadly speaking, fission promotes the existence of a sufficient mitochondrial pool size to meet cellular energy demand, while fusion maintains a functional mitochondrial network (52). Consequently, we surveyed the expression of key genes involved in mitochondrial homeostasis and known to be transcriptionally modulated by HFDs in rodent models. p62/sequestosome 1 (p62) is degraded during autophagy (51, 53). Consistently, p62 accumulates in autophagy-deficient cells and in HFD-exposed organs (54, 55, 56, 57). By contrast, expression of fission-promoting dynamin 1 like (DRP1) and fission, mitochondrial 1 (FIS1) are decreased by HFD (54). Expression of *p62* and *Drp1* change in opposite directions in pathological conditions in the liver and skeletal muscle, where loss of DRP1 is detrimental and decreases FA oxidation (44, 58, 59). Accordingly, biochemical inhibition of fission did not correct steatosis in a mouse model of fatty liver (60). Despite that consistent evidence, the association between fission and disease may be tissue-specific, as the inhibition of fission moderated the effects of hypertension in the circulatory system (61). As for mitochondrial fusion, expression of the profusion mitofusin 2 (MFN2) is decreased in cultured cells exposed to high-fat medium, whereas ectopic MFN2 expression mitigates the adverse metabolic effects of an HFD in the mouse liver (62, 63).OPA1 is another profusion protein that to our knowledge has been surveyed only in one mammalian model of HFD (64, 65).

We first assessed the effects of HAFPD on mitochondrial function-related gene expression, by comparison with chow-fed mice. *p62* expression was significantly higher in 8WVeh than chow-fed counterparts (58.3 ± 7.1 and 17.7 ± 1.5, respectively (all expression data are expressed as 2^-ΔCt^ relative to *Gapdh*); *P* = 7.4 × 10^−5^). That trend was maintained in 16W (1.4 ± 1.0 and 0.2 ± 0.1, respectively, *P* = 0.011). At the same time, *p62* expression was markedly decreased from 8WVeh to 16WVeh; that decrease was driven by either duration of feeding with HAFPD or age or by a combination thereof, as *p62* expression markedly decreased in chow-fed controls over a period corresponding to 8W and 16W (17.7 ± 1.5 and 0.2 ± 0.1, *P* = 7.8×10^−7^) (Fig. 5*C*). By contrast, the expression of transcripts encoding profission proteins was dramatically increased in 8WVeh relative to chow-fed controls: *Drp1*, 4.2 ± 1.1 and 22.3 ± 2.2, respectively, or ∼5.5-fold, *P* = 9.1 × 10^−6^; *Fis1*, 0.3 ± 0.1 and 6.2 ± 1.5, respectively, or ∼23-fold, *P* = 8.8 × 10^−7^. In 16W, the HAFPD-induced decline of profission gene expression compared to chow-fed controls was maintained although it was relatively modest for *Drp1* (17.0 ± 4.1 and 31.1 ± 4.4, *P* = 0.021) but robust for *Fis1* (0.1 ± 0.1 and 41.4 ± 11.6, *P* = 9.0 × 10^−8^). *Drp1* expression was higher in 16WVeh than 8WVeh (Fig. 5*D*); that trend was mainly due to duration of feeding with HAFPD, as the difference between 8W-matched and 16W-matched was weak in chow-fed mice (22.3 ± 2.2 and 31.1 ± 4.4, *P* = 0.062). Similarly, the lower *Fis1* expression in 16WVeh than 8WVeh was due to duration of HAFPD, as chow-fed controls showed the opposite trend (6.2 ± 1.5 in 8W, 41.4 ± 11.6 in 16W, *P* = 0.001) (Fig. 5*E*). As for *Mfn2* expression, no significant change was observed between 8WVeh and 8WDec or between 16wVeh and 16WDec (lowest *P* = 0.451). The decrease in *Mfn2* expression between 8WVeh and 16WVeh was likely age-driven, as a similar decrease was observed in chow-fed mice between time points corresponding to 8W and 16W (∼2-fold in HAFPD-fed mice, ∼3.1-fold in chow-fed mice, *P* < 0.01) (Fig. 5*F*). *Opa1* expression was highly variable and undetectable in most samples.

In turn, *p62* expression was decreased, but *Drp1* and *Fis1* expression was increased in 8WDec compared with 8WVeh, with marked differences in all cases (∼2- to 9-fold range); those effects were maintained in 16W for *p62* and *Fis1*, but not *Drp1* (Fig. 5, *C–E*). Duration of feeding with HAFPD overrun any effect of Dec on *Drp1* expression by realigning 16WVeh and 16WDec, as the decrease in *Drp1* expression between 8WDec and 16WDec ran opposite to the weak increase between marched chow-fed controls (12.8 ± 2.2 and 18.0 ± 3.1, *P* = 0.056) (Fig. 5*D*). No significant effect of Dec was observed in the case of the profusion *Mfn2* gene (Fig. 5*F*). No meaningful data could be obtained for *Opa1*, as expression was highly variable and undetectable in most samples.

### Effects of Dec on whole blood acylcarnitines

**Fig. 6:**
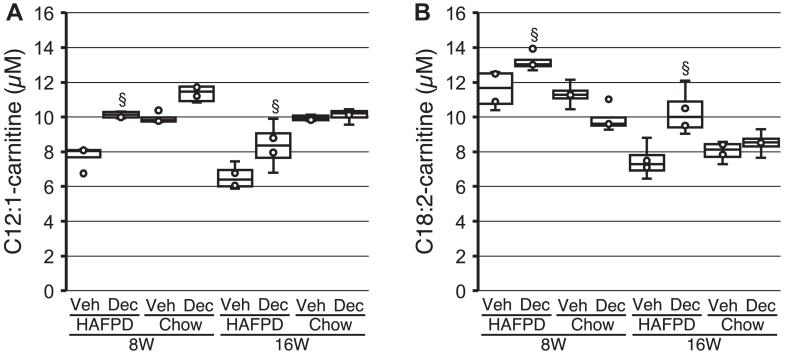
Effects of decitabine on representative whole blood acylcarnitines in high-animal fat and protein diet (HAFPD)-fed female mice. Box and whisker plots of data from HAFPD-fed or chow-fed mice exposed to vehicle or decitabine (Veh or Dec, respectively). Significance refers to comparisons with the respective control (Veh). §, *P* < 0.1. Kruskal–Wallis and ANOVA Scheffé’s post hoc. A and B: representative acylcarnitines of group 1 and 2, respectively. N = 4/group.

If Dec ameliorates mitochondrial functionality, it is expected to maintain physiological levels of acylcarnitines, which are intermediates of mitochondrial uptake of FAs destined to beta-oxidation (66). To gain insights into the global effects of HAFPD and Dec, we surveyed free carnitine and 34 short, medium, and long acylcarnitine species in whole blood. In 8W, two distinct acylcarnitine profiles were identified. A set of seven short, medium, and long acylcarnitines (C3OHC, C5C, C5:1C, C5OHC, C8C, C10C, and C12:1C) were lower in HAFPD-fed 8WVeh than matched chow-fed controls, although differences reached borderline significance (0.1 < *P* < 0.05). Those acylcarnitines were not different from chow-fed controls in 8WDec (Fig. 6*A* and supplemental Table S4). This acylcarnitine set will be referred to as group 1. A second set of four long acylcarnitines (C16OHC, C16:1OHC, C16:2OHC, and C18:2C) were not significantly different between HAFPD-fed 8WVeh and matched chow-fed controls, but tended to be elevated in 8WDec, in comparison with 8WVeh or chow-fed mice. This acylcarnitine set will be referred to as group 2 (Fig. 6*B* and supplemental Table S4). Fifty percent of the identified acylcarnitines (two out of seven in group 1; four out of four in group 2) retained a memory of past exposure to Dec, that is, showed similar profiles in 8W and 16W (Fig. 6, *A* and *B* and supplemental Table S4).

## Discussion

We confirm the original hypothesis on the primary outcome, that is, Dec slowed BW gain although markedly in females and only marginally in male mice. Also, Dec ameliorated HAFPD-induced lipid accumulation in the liver. In the skeletal muscle, Dec increased lipid content and transcriptional markers of mitochondrial functionality. These changes were accompanied by an increase of circulating acylcarnitines, another marker of mitochondrial functionality, and NEFA. The increase in NEFA coincided with increased adipose-IR, suggesting augmented adipose tissue lipolysis and availability of FA for skeletal muscle mitochondrial oxidation. Adipose-IR was induced by both Dec and HAFPD (see below), implying that Dec additionally favors FA uptake by the skeletal muscle. The results are consistent with our initial hypothesis that DNA hypermethylation accompanies and is a causal mediator of at least part of the pathophysiological drift caused by exposure to CVD risk factors such as HFD.

HAFPD induced a range of traits previously observed in animal models of HFD: increased BW and hepatic lipids, altered mitochondrial function–related gene expression, and a tendency to decrease selected acylcarnitines, although the latter response was weak likely due to lack of statistical power (54, 67, 68). Additionally, HAFPD induced adipose-IR, but not HOMA-IR. The two indexes were not correlated in our samples. Our data are consistent with previous observations that adipose-IR is dissociated from systemic insulin resistance (69). The extent of the HAFPD-induced increase in NEFA was comparable with the corresponding changes observed in human fatty liver or type 2 diabetes (41, 70). Dec generally counteracted the effects of HAFPD. Our data echo the previously observed mild decrease in BW induced by the Dec analog azacytidine in chow-fed mice (37). Additionally, we highlight noteworthy effects of Dec. First, we observed an apparently paradoxical profile of skeletal muscle mitochondrial DNA content, which was increased by either Dec or duration of feeding with HAFPD. Based on tissue fat content and gene expression data, we propose the following model to reconcile that apparent contradiction: HAFPD maintains defective mitochondria by lowering autophagy and slowing mitochondrial fission; in turn, Dec increases lipid demand by promoting mitochondrial quality control and functionality through autophagy and fission; a mismatch between lipolysis and lipid influx or de novo lipogenesis rate would explain the observed increase in skeletal muscle lipid accumulation. We further submit that by increasing adipose-IR, Dec increases the pool of circulating FAs available for skeletal muscle metabolism. The resulting increase in energy expenditure would account for Dec-induced decrease of BW gain. As noted above, that phenotype may resemble the human phenomenon known as "athlete's paradox" (49, 50). Additionally, Dec may promote lipolysis by adipocyte browning, akin to the Dec analog azacytidine (71). Interestingly, we reproduced previous observations that Dec and azacytidine increase lipid droplet formation in murine embryonic fibroblasts and in cancer cell lines (72, 73, 74). Whether those models mechanistically resemble our in vivo findings, particularly from the viewpoint of mitochondrial functionality and regulation of endogenous lipogenesis, cannot be concluded based on available data. At any rate, our data echo the reported liver fat–lowering response to azacytidine in a murine model of diet-induced fatty liver (8).

The antagonism between HAFPD and Dec is clear for 8W, but 16W data suggest a complex balance between memory of past exposure to Dec and duration of HAFPD feeding. In the case of tissue fat content, memory of past exposure to Dec was strong in liver and skeletal muscle, with no detectable consequence of either duration of feeding with HAFPD or age. By contrast, other traits showed a tissue-specific effect of HAFPD on the memory of previous exposure to Dec. Mitochondrial DNA content and global DNA methylation were decreased and increased, respectively, in either liver or skeletal muscle that were continuously exposed to Dec for 8 weeks, but a memory of previous—8 weeks earlier—exposure to Dec was unequivocal only in the liver. As *caveat*, it is likely that functionally important genes retain epigenetic memory of exposure to Dec but are overlooked in our study if their contribution to global DNA methylation is modest. In the skeletal muscle, the apparent loss of memory of previous exposure to Dec was due to a prominent effect of HAFPD, which increased mitochondrial DNA content in 16WVeh to the extent that 16WVeh and 16WDec were realigned. A similar pattern was apparent in adipose tissue mitochondrial DNA content, although we could not appreciate the effect of ageing per se in that case. By contrast, memory of previous exposure to Dec was weak for all analyzed mitochondria-related transcripts, likely due to gene-specific mechanisms: in the case of *p62*, memory of exposure to Dec persisted, but HAFPD and age, either individually or in combination, imposed a marked decline in expression in 16W; *Drp1* expression was realigned in 16W by duration of feeding with HAFPD, with a minor effect of ageing if any; in turn, *Fis1* expression was lowered by prolonged feeding with HAFPD, apparently overrunning any effect of Dec. Therefore, 16W data suggest that duration of feeding with HAFPD exacerbated the decline of *Fis1* expression but apparently sustained autophagy. This partially beneficial effect of prolonged HAFPD feeding may contribute to the tendency of BW gain to reach a plateau between 8W and 16W. Also, the fact that skeletal muscle lipid content retained a strong memory of previous exposure to Dec suggests that Dec imposed stable changes of the mitochondrial pool or NEFA uptake or both, which continued to shape tissue lipid content, despite the overriding effects of the duration of HAFPD feeding or age on mitochondria-related gene expression or plasma NEFA, respectively. That scenario is supported by the observation that selected acylcarnitines retain a memory of previous Dec exposure. A likely factor affecting the response to Dec is the timing of change in mouse body composition: body mass index and fat mass index are highly dynamic between the age of 5 and 17 weeks (75). 8W (14 weeks of age) falls within that age range, whereas in 16W (22 weeks of age) adipose tissue accumulation reaches a plateau. Therefore, it can be speculated that transcription of relevant *loci* is more sensitive to Dec in 8W, compared to the relatively "locked" state of 16W. As for liver weight, the contrasting effects of Dec in 8W and 16W (decrease and increase, respectively, relative to feeding with HAFPD only) may represent an effort to strengthen the organ's lipolytic capacity, although increased cellularity or fibrosis cannot be ruled out. Taken together, our results are compatible with the reported widely variable degree of transcriptional memory of exposure to Dec or azacytidine: the effect of azacytidine is markedly gene-specific and a clear memory was observed only of genes that are strongly demethylated by the original exposure to azacytidine in cultured cells (76); memory of previous exposure to azacytidine is dose-dependent and partial, corresponding to ∼10–50% of maximal azacytidine-induced global DNA demethylation, in HEK 293T cells (77). The partial memory to a previously imposed epigenetic modification is likely due mechanisms of transcriptional regulation that are additional to DNA methylation, as suggested by the incomplete reversion of BW and adiposity to chow-fed control levels, in high-fat diet-fed DNA methyltransferase–null mice (78). By contrast, cellular phenotypes induced by Dec or azacytidine in cultured cells can persist after weeks of subsequent growth in drug-free medium or successive xenograft transplantations to unexposed mouse recipients (79, 80).

Additionally, we observed a marked sexual dimorphism. Our data mirror previous evidence that in general male mice display more extreme responses to metabolic challenge and are resistant to corrective interventions compared to females. Examples of such traits in males compared to females are as follows: higher cardiometabolic risk markers following feeding with high-fat or specific FA-rich diets (81, 82); lower circulating adiponectin, a trait which we confirmed in the present study (data not shown) (83); marked resistance to genetic modification that mitigates obesity or promotes adipose tissue browning (78, 84). As for sexual dimorphism in responses to DNA methylation inhibitors, data are relatively scarce. One study indicates that an HFD induces DNA hypomethylation in male rats, but the opposite response in females (85). Also, the loss of DNA methyltransferase DNMT3a in hematopoietic cells affected the response to an HFD in males, but not in female mice (12). Although the molecular details of the dimorphic response in that model are unknown, it can be speculated that crucial *loci* are constitutively hypomethylated in male mice and therefore their expression is insensitive to Dec; additionally, those *loci* would be relevant for females but redundant in males due to compensating male-specific signaling pathways. Furthermore, sexual dimorphism may be dependent on the specific developmental phase of intervention, as male mice are more sensitive to exposure to azacytidine in utero compared to females (86). Also, azacytidine induces favorable behavioral effects in the early postnatal life in either sex but the affected behaviors are sexually dimorphic (87). These observations are relevant from a medical viewpoint, as sexual dimorphism in the propensity to metabolic disorders is well documented in humans (88, 89).

In summary, our results strengthen the idea that DNA hypermethylation plays a pivotal role in the onset and progression of metabolic disease and CVD. Thus, efforts are warranted to explore the feasibility of safe and effective DNA methylation control in primary prevention. The latter concerns are underlined by the *caveat* that Dec induced adipose-IR in our model. An essential requirement for any clinical application is that DNA methylation is manipulated in a highly target tissue-specific fashion to avoid indiscriminate drifts from the epigenome's physiological profile. From a basic science viewpoint, further experimental work is needed to reveal the details of the regulation of cellular metabolism and DNA methylome by Dec in our and related models.

## Data availability

Does not apply.

## Supplemental data

This article contains supplemental data.

## Conflict of interest

The authors declare that they have no conflicts of interest with the contents of this article.
