# Supplementary material for: The DNA methyltransferase inhibitor decitabine blunts the response to a high-animal fat and protein diet in mice
Source: J Lipid Res. 2024 Jun 26;65(8):100586. doi: 10.1016/j.jlr.2024.100586 (PMC11325794; doi:10.1016/j.jlr.2024.100586)
Supplement: Supplementary material [file mmc1.pdf]

Supplemental material

Manuscript title: **The DNA methyltransferase inhibitor decitabine blunts the response to a high animal fat and protein diet in mice**

First author's surname: **Flores-Sierra**

List of supplemental figures and tables:

Figure S1

Figure S2

Figure S3

Table S1

Table S2

Table S3

Table S4

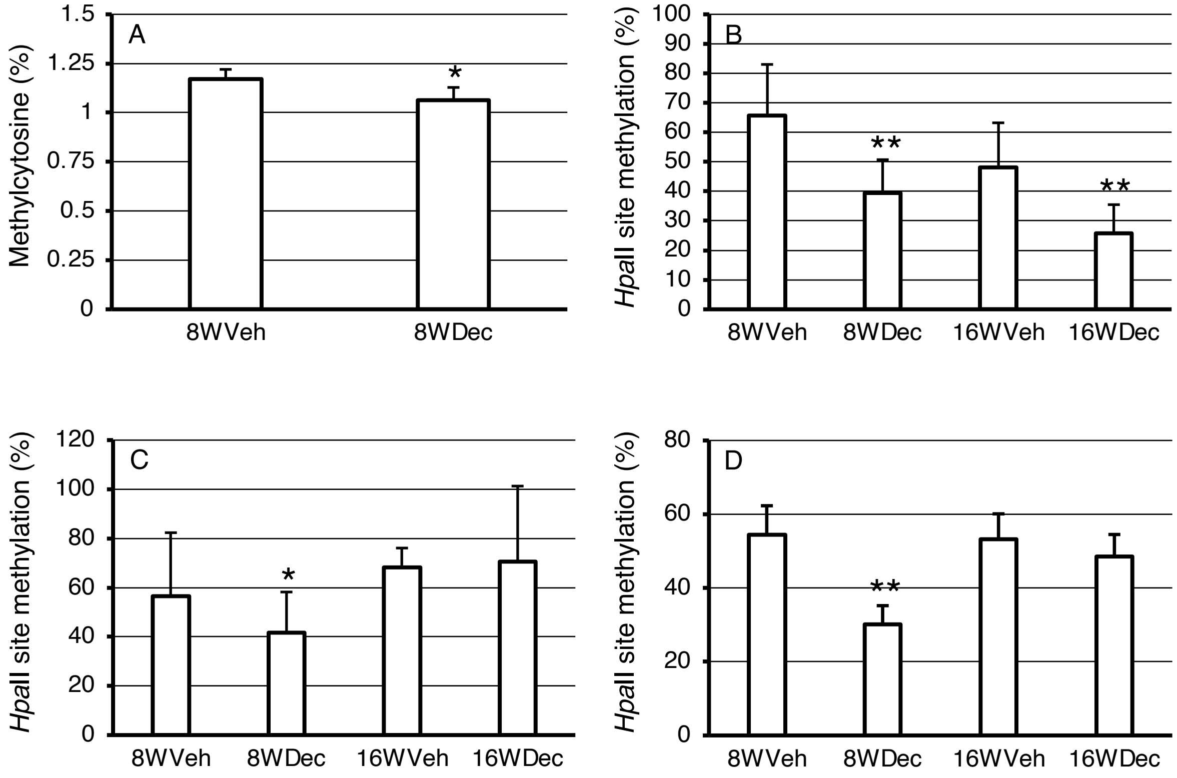

Fig. S1. **Effects of Dec on global DNA methylation.** *A*, ELISA-based determination of methylcytosine. *B-D*, fluorescence-based determination of methylated *Hpa*II sites. *A-B*, liver; *C*, skeletal muscle; *D*, adipose tissue. Data are mean±SEM. n=4/group. 8W, groups exposed to high-animal fat and high-animal protein diet (HAFPD), and to either decitabine (Dec) or vehicle (Veh) for 8 weeks. 16W, groups exposed to HAFPD for 16 weeks, in addition to Dec or Veh exposure during the first eight weeks. Asterisks refer to significance of comparisons with corresponding Veh controls. *, $p<0.05$. **, $p<0.01$. ANOVA and Scheffé's *post hoc*.

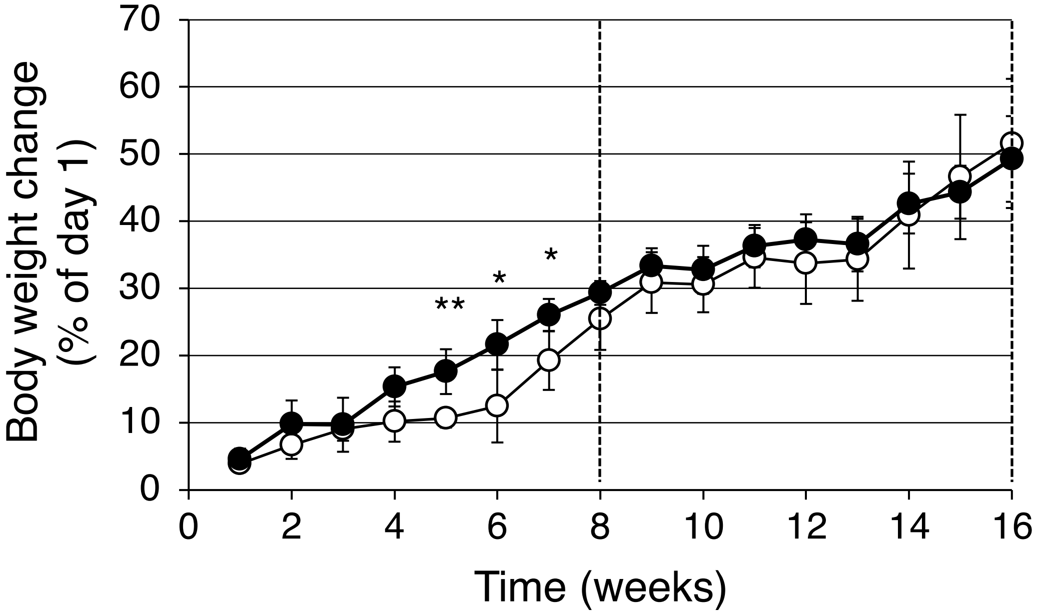

Fig. S2. **Effects of decitabine on body weight in high animal fat and protein diet (HAFPD)-supplemented male mice.** Empty and solid circles, Dec-exposed and vehicle-exposed mice, respectively. Data are mean±SEM. n=5 per group. The vertical dashed lines mark the time of sacrifice of mice with continuous exposure to Dec for 8 weeks (8W set, left-hand dashed line; n=22 combining 12 8W and 10 16W) or with Dec withdrawal at week 8 (16W set, right-hand dashed line; n=10). Asterisks above time points indicate significant differences between Dec-exposed and vehicle-exposed HAFPD-fed mice. **, $p<0.01$. ***, $p<0.001$. Kruskal-Wallis test and ANOVA Scheffé's *post hoc*.

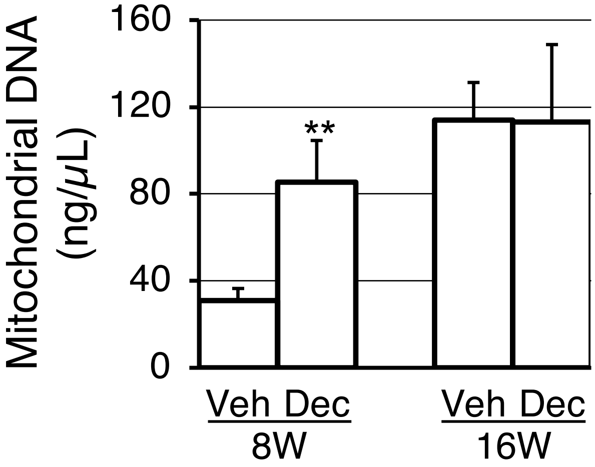

Fig. S3. **Effects of Dec on adipose tissue mitochondrial DNA content.** 8W, groups exposed to high-animal fat and high-animal protein diet, and to either Dec or vehicle (Dec and veh, respectively) for 8 weeks. 16W, groups exposed to high-animal fat and high-animal protein diet only, and previously exposed to either Dec or vehicle (Dec and Veh, respectively) for further 8 weeks (16 weeks in total). Data are mean$\pm$SEM. N=10/group. Asterisks refer to significance of comparisons with the corresponding vehicle controls. *, $p<0.05$. **, $p<0.01$. ANOVA and Scheffé's *post hoc*.

Table S1. **Composition of high animal fat and protein diet (HAFPD) and chow.**

Component	HAFPD	Chow (LabDiet 5001)
Protein[a]	32.00	23.90
Fat[a]	20.00	5.00
Fatty acids[b]:		
C12:0	0.03	0.00
C14:1	0.11	0.00
C14:0	1.72	0.85
C15:0	0.18	0.04
*iso*methyl-C15:0	0.08	0.00
n-9 C16:1	0.29	0.10
n-7 C16:1	3.38	1.50
C16:0	23.52	22.02
*iso*methyl-C16:0	0.11	0.00
C17:1	0.30	0.10
C17:0	0.65	0.23
C18:3	0.25	0.10
C18:2	18.88	25.96
cis C18:1	29.05	34.78
trans C18:1	3.04	2.23
C18:0	16.72	10.57
C20:4	0.43	0.12
C20:5	0.03	0.18
n-9 C20:3	0.00	0.00
n-6 C20:3	0.08	0.00
C20:2	0.33	0.25
C20:1	0.58	0.48
C20:0	0.25	0.20
C20:6	0.00	0.29
SFA	43.26	33.91
MUFA	36.75	39.19
PUFA	19.99	26.90
Fiber[a]	3.00	5.10
Humidity[a]	12.00	17.00
Ash[a]	7.50	7.00
Calcium[a]	1.00 - 1.40	0.90
Phosphorus[a]	0.90 - 1.30	0.70

[a]Percent (w/w). [b]Percent of total fatty acids (w/w).

Table S2. **Administered decitabine absolute dose.**

Week	Females, HAFPD	Females, chow	Males, HAFPD
0	16.9	17.2	20.7
1	17.8	17.2	21.5
2	18.6	18.0	22.1
3	19.4	19.2	22.6
4	20.2	19.9	22.8
5	20.8	20.9	22.9
6	20.9	21.2	23.3
7	21.4	22.7	24.7
8	21.9	22.6	26.0
9	22.5	23.4	27.1
10	23.5	23.5	27.0
11	23.7	24.1	27.9
12	23.8	23.8	27.7
13	24.3	24.0	27.8
14	24.7	24.6	29.2
15	24.9	25.2	30.3
Total	345.5	330.3	403.5

Average µg administered decitabine is shown.

Table S3. **NEFA raw data. (µg/50 µL plasma).**

Treatment	Time/diet	C14:0	C16:1 7Z	C16:1 9Z	C16:0	C17:0	C18:3 (9,12,15Z)	C18:2 (9,12Z)	C18:1 (9Z)	C18:1 (9E)	C18:0	C20:0
Veh	8W chow	0.2	0.0	0.3	4.1	0.0	0.0	0.7	1.0	0.3	3.7	0.2
Veh	8W chow	0.2	0.0	0.2	1.4	0.0	0.0	0.5	0.7	0.3	1.4	0.2
Veh	8W chow	0.2	0.0	0.2	3.1	0.0	0.0	0.3	0.6	0.3	2.4	0.2
Veh	8W chow	0.2	0.0	0.2	3.1	0.0	0.0	0.3	0.5	0.3	2.3	0.2
Veh	8W chow	0.0	0.0	0.2	1.4	0.0	0.0	0.2	0.4	0.3	1.3	0.2
Veh	8W chow	0.2	0.0	0.2	2.7	0.0	0.0	0.2	0.4	0.3	2.0	0.2
Veh	8W chow	0.2	0.2	0.2	1.7	0.0	0.0	0.7	0.9	0.3	2.1	0.2
Veh	8W chow	0.0	0.0	0.2	2.8	0.0	0.0	0.3	0.6	0.3	2.2	0.2
Veh	8W chow	0.2	0.2	0.2	2.1	0.0	0.0	0.6	0.7	0.3	1.9	0.2
Veh	8W chow	0.0	0.0	0.2	3.4	0.0	0.0	0.4	0.6	0.3	2.8	0.2
Veh	8W HAFPD	0.2	0.0	0.2	6.5	0.0	1.1	0.5	0.3	0.3	4.2	0.2
Veh	8W HAFPD	0.2	0.0	0.0	6.5	0.0	0.4	0.3	0.3	0.2	4.8	0.2
Veh	8W HAFPD	0.0	0.0	0.2	3.2	0.0	0.3	0.6	0.6	0.3	3.6	0.2
Veh	8W HAFPD	0.2	0.0	0.0	5.4	0.0	0.2	0.5	0.4	0.2	4.1	0.2
Veh	8W HAFPD	0.2	0.0	0.2	4.8	0.0	0.0	0.6	0.6	0.3	3.9	0.2
Veh	8W HAFPD	0.2	0.0	0.2	6.8	0.0	0.0	0.3	0.4	0.2	5.1	0.2
Veh	8W HAFPD	0.2	0.2	0.3	4.5	0.0	0.0	0.8	0.9	0.3	3.9	0.2
Veh	8W HAFPD	0.2	0.0	0.2	8.0	0.0	0.0	0.5	0.5	0.3	5.9	0.2
Veh	8W HAFPD	0.3	0.2	0.2	4.5	0.0	0.0	0.6	0.8	0.3	4.0	0.2
Veh	8W HAFPD	0.3	0.2	0.2	7.8	0.0	0.0	0.5	0.6	0.4	5.8	0.2
Dec	8W chow	0.0	0.0	0.0	5.9	0.0	0.0	0.0	0.4	0.3	4.2	0.2
Dec	8W chow	0.2	0.0	0.2	5.8	0.0	0.0	0.2	0.4	0.3	4.4	0.2
Dec	8W chow	0.0	0.0	0.0	4.5	0.0	0.0	0.0	0.3	0.3	3.4	0.2
Dec	8W chow	0.0	0.0	0.0	4.5	0.0	0.0	0.0	0.4	0.3	3.7	0.2
Dec	8W chow	0.0	0.0	0.0	3.8	0.0	0.0	0.0	0.3	0.3	3.2	0.2
Dec	8W chow	0.0	0.0	0.2	2.6	0.0	0.0	0.3	0.7	0.3	3.4	0.2
Dec	8W chow	0.2	0.0	0.2	6.7	0.0	0.0	0.3	0.5	0.3	4.8	0.2
Dec	8W chow	0.0	0.0	0.2	3.3	0.0	0.0	0.6	0.6	0.3	3.9	0.2
Dec	8W chow	0.2	0.0	0.2	5.4	0.0	0.0	0.6	0.7	0.3	4.5	0.2
Dec	8W chow	0.2	0.0	0.2	4.7	0.0	0.0	0.4	0.9	0.3	5.3	0.2
Dec	8W HAFPD	0.0	0.0	0.3	4.3	0.0	0.0	0.6	1.1	0.3	4.0	0.2
Dec	8W HAFPD	0.2	0.0	0.2	4.1	0.0	0.0	0.7	1.1	0.3	3.6	0.2
Dec	8W HAFPD	0.2	0.0	0.3	6.0	0.0	0.0	0.5	0.9	0.3	4.7	0.2
Dec	8W HAFPD	0.2	0.0	0.2	4.9	0.0	0.0	0.6	0.9	0.3	5.0	0.2
Dec	8W HAFPD	0.3	0.0	0.2	11.1	0.0	0.0	0.4	1.1	0.3	6.5	0.2
Dec	8W HAFPD	0.0	0.0	0.2	6.1	0.0	0.0	0.3	0.5	0.3	4.4	0.2
Dec	8W HAFPD	0.2	0.0	0.2	8.0	0.2	0.0	0.0	0.4	0.3	5.4	0.2
Dec	8W HAFPD	0.2	0.0	0.2	8.2	0.0	0.0	0.0	0.4	0.3	5.5	0.2
Dec	8W HAFPD	0.2	0.0	0.0	7.6	0.0	0.0	0.0	0.3	0.3	5.4	0.2
Dec	8W HAFPD	0.2	0.0	0.0	10.4	0.0	0.0	0.0	0.3	0.3	7.3	0.2
Veh	16W chow	0.0	0.0	0.0	5.2	0.0	0.0	0.7	0.7	0.3	5.7	0.2
Veh	16W chow	0.2	0.0	0.2	5.3	0.0	0.0	0.9	1.0	0.4	4.5	0.2
Veh	16W chow	0.2	0.0	0.2	11.1	0.2	0.0	0.6	0.9	0.3	7.7	0.2
Veh	16W chow	0.2	0.0	0.2	9.3	0.0	0.0	0.4	0.7	0.3	6.2	0.2
Veh	16W chow	0.0	0.0	0.0	4.8	0.0	0.0	0.6	0.8	0.4	5.3	0.2
Veh	16W chow	0.2	0.0	0.3	4.8	0.0	0.0	0.9	1.1	0.5	4.8	0.2
Veh	16W chow	0.2	0.0	0.2	7.9	0.0	0.0	0.5	1.0	0.3	5.4	0.2
Veh	16W chow	0.2	0.0	0.2	5.4	0.0	0.0	0.6	0.9	0.3	4.4	0.2
Veh	16W chow	0.0	0.0	0.2	4.9	0.0	0.0	0.7	1.0	0.4	4.1	0.2
Veh	16WHAFPD	0.2	0.0	0.2	8.4	0.0	0.0	0.4	0.9	0.3	5.9	0.2
Veh	16WHAFPD	0.2	0.0	0.2	6.4	0.0	0.0	0.5	0.8	0.4	4.1	0.2
Veh	16WHAFPD	0.2	0.0	0.3	7.0	0.2	0.0	0.5	1.0	0.4	5.2	0.2
Veh	16WHAFPD	0.2	0.0	0.2	8.2	0.0	0.0	0.4	0.7	0.4	5.6	0.2
Veh	16WHAFPD	0.3	0.0	0.2	5.9	0.2	0.0	0.7	1.1	0.5	5.6	0.2
Veh	16WHAFPD	0.2	0.0	0.3	9.3	0.2	0.0	0.5	0.9	0.4	6.7	0.2
Veh	16WHAFPD	0.2	0.0	0.2	6.1	0.2	0.0	0.7	0.9	0.4	6.0	0.2
Veh	16WHAFPD	0.2	0.0	0.2	11.8	0.2	0.0	0.6	1.0	0.4	7.9	0.2
Veh	16WHAFPD	0.2	0.0	0.2	5.3	0.0	0.0	0.4	1.0	0.3	5.5	0.2
Veh	16WHAFPD	0.2	0.0	0.2	12.3	0.2	0.0	0.3	0.8	0.4	8.2	0.2
Dec	16W chow	0.3	0.0	0.2	13.3	0.2	0.0	0.5	0.6	0.3	9.3	0.2
Dec	16W chow	0.2	0.0	0.2	11.2	0.2	0.0	0.2	0.5	0.4	7.9	0.2
Dec	16W chow	0.2	0.0	0.2	10.1	0.0	0.0	0.0	0.5	0.3	6.7	0.2
Dec	16W chow	0.2	0.0	0.2	10.5	0.0	0.0	0.2	0.4	0.3	6.8	0.2
Dec	16W chow	0.3	0.0	0.2	12.7	0.2	0.0	0.0	0.4	0.4	8.8	0.2
Dec	16W chow	0.2	0.0	0.2	6.4	0.0	0.0	0.4	0.5	0.3	4.4	0.2
Dec	16W chow	0.3	0.0	0.2	14.1	0.2	0.0	0.3	0.5	0.3	9.5	0.2
Dec	16W chow	0.2	0.0	0.2	6.8	0.0	0.0	0.6	0.6	0.3	4.8	0.2
Dec	16W chow	0.2	0.0	0.2	8.1	0.0	0.0	0.6	0.8	0.3	5.4	0.2
Dec	16W chow	0.2	0.0	0.2	6.5	0.0	0.0	0.6	0.9	0.3	4.8	0.2
Dec	16WHAFPD	0.2	0.0	0.2	6.9	0.2	0.0	0.9	1.3	0.4	6.7	0.3
Dec	16WHAFPD	0.2	0.0	0.2	5.5	0.2	0.0	0.6	1.0	0.5	3.4	0.2
Dec	16WHAFPD	0.2	0.0	0.2	5.8	0.2	0.0	0.6	1.1	0.4	4.2	0.2
Dec	16WHAFPD	0.2	0.0	0.2	6.4	0.2	0.0	0.4	1.2	0.5	4.2	0.2
Dec	16WHAFPD	0.2	0.0	0.2	10.0	0.2	0.0	0.3	0.9	0.3	6.4	0.2
Dec	16WHAFPD	0.2	0.0	0.2	9.7	0.0	0.0	0.2	0.7	0.4	6.1	0.2
Dec	16WHAFPD	0.2	0.0	0.2	7.7	0.0	0.0	0.0	0.5	0.3	5.2	0.2
Dec	16WHAFPD	0.2	0.0	0.2	9.6	0.0	0.0	0.2	0.5	0.3	6.4	0.2
Dec	16WHAFPD	0.2	0.0	0.2	8.4	0.0	0.0	0.0	0.4	0.4	5.3	0.2
Dec	16WHAFPD	0.3	0.0	0.2	8.3	0.0	0.0	0.2	0.4	0.4	5.7	0.2

Veh and Dec, vehicle and decitabine, respectively. 8W and 16W, groups terminating Dec exposure at week 8 and week 16, respectively. HAFPD, high animal fat and protein diet. NEFA are indicated by conventional nomenclature: number of carbons:number of carbon-carbon double bounds (position of double bounds). Z and E, *cis* and *trans* configuration of double bonds, respectively.

Table S4. **Effect of decitabine on selected whole blood acylcarnitine in high animal fat and protein diet-fed mice.**

		8W			16W		
	Acyl-carnitine	HAFPD/ chow	HAFPD+Dec/ HAFPD	HAFPD+Dec/ chow	HAFPD/ chow	HAFPD+Dec/ HAFPD	HAFPD+Dec/ chow
Group 1	C12:1C	0.79§	1.37§	1.08	0.71§	1.28§	0.90
	C10C	0.28§	2.43§	0.97	0.69§	1.14	0.80
	C8C	0.80§	1.32§	1.05	0.72§	2.0§	1.04
	C5C	0.67§	1.20§	0.80	0.60§	1.14	0.75
	C5OHC	0.66§	1.32§	0.86	1.15	0.86	0.86
	C5:1C	0.43§	1.85§	0.79	0.48§	0.94	0.49
	C3OHC	0.50§	1.39§	0.69	1.26	1.75	0.78
Group 2	C18:2C	0.97	1.32§	1.24§	1.00	1.33§	1.33§
	C16OHC	1.03	1.39§	1.39§	1.03	1.27§	1.29§
	C16:1OHC	1.08	1.33§	1.81§	1.12	1.34§	1.56§
	C16:2OHC	1.05	1.37§	1.35§	0.98	1.42§	1.39§

Acylcarnitines are indicated as standard annotation of the corresponding fatty acid - number of carbons, degree of saturation and hydroxylation if any - followed by a C for carnitine. 8W, HAFPD-fed mice exposed to vehicle or Dec for eight weeks. 16W, mice treated as 8W but exposed to HAFPD alone for further 8 weeks. Dec, decitabine. HAFPD, high animal fat and protein diet. §, $0.1>p>0.05$.
